# Detection of Breeding-Relevant Fruit Cracking and Fruit Firmness Quantitative Trait Loci in Sweet Cherry *via* Pedigree-Based and Genome-Wide Association Approaches

**DOI:** 10.3389/fpls.2022.823250

**Published:** 2022-03-02

**Authors:** William Wesley Crump, Cameron Peace, Zhiwu Zhang, Per McCord

**Affiliations:** ^1^Department of Horticulture, Washington State University, Prosser, WA, United States; ^2^Department of Horticulture, Washington State University, Pullman, WA, United States; ^3^Department of Crop and Soil Sciences, Washington State University, Pullman, WA, United States

**Keywords:** *Prunus avium* L., cracking, firmness, breeding, genome-wide association study, pedigree-based analysis, FlexQTL, BLINK

## Abstract

Breeding for decreased fruit cracking incidence and increased fruit firmness in sweet cherry creates an attractive alternative to variable results from cultural management practices. DNA-informed breeding increases its efficiency, yet upstream research is needed to identify the genomic regions associated with the trait variation of a breeding-relevant magnitude, as well as to identify the parental sources of favorable alleles. The objectives of this research were to identify the quantitative trait loci (QTLs) associated with fruit cracking incidence and firmness, estimate the effects of single nucleotide polymorphism (SNP) haplotypes at the detected QTLs, and identify the ancestral source(s) of functional haplotypes. Fruit cracking incidence and firmness were evaluated for multiple years on 259 unselected seedlings representing 22 important breeding parents. Phenotypic data, in conjunction with genome-wide genotypic data from the RosBREED cherry 6K SNP array, were used in the QTL analysis performed *via* Pedigree-Based Analysis using the FlexQTL™ software, supplemented by a Genome-Wide Association Study using the BLINK software. Haplotype analysis was conducted on the QTLs to identify the functional SNP haplotypes and estimate their phenotypic effects, and the haplotypes were tracked through the pedigree. Four QTLs (two per trait) were consistent across the years and/or both analysis methods and validated the previously reported QTLs. qCrack-LG1.1m (the label given to a consistent QTL for cracking incidence on chromosome 1) explained 2–15.1% of the phenotypic variance, while qCrack-LG5.1m, qFirm-LG1.2m, and qFirm-LG3.2m explained 7.6–13.8, 8.8–21.8, and 1.7–10.1% of the phenotypic variance, respectively. At each QTL, at least two SNP haplotypes had significant effects and were considered putative functional SNP haplotypes. Putative low-cracking SNP haplotypes were tracked to an unnamed parent of ‘Emperor Francis’ and ‘Schmidt’ and unnamed parents of ‘Napoleon’ and ‘Hedelfingen,’ among others, and putative high-firmness haplotypes were tracked to an unnamed parent of ‘Emperor Francis’ and ‘Schmidt,’ an unnamed grandparent of ‘Black Republican,’ ‘Rube,’ and an unknown parent of ‘Napoleon.’ These four stable QTLs can now be targeted for DNA test development, with the goal of translating information discovered here into usable tools to aid in breeding decisions.

## Introduction

Two attributes sought after by sweet cherry (*Prunus avium* L.) breeding programs are tolerance to rain-induced fruit cracking (hereafter referred to as “cracking incidence” or simply “cracking”) and high fruit firmness (Quero-Garcia et al., 2017). Cracking is the most severe abiotic threat to profitability in sweet cherries (Quero-Garcia et al., 2017), especially in areas of production where rain events are common near harvest. Higher labor costs during harvest and in packing facilities from roguing cracked fruits mean that a relatively small proportion of cracked fruits (Looney, 1985 estimated more than about 25%) can lead to an unprofitable harvest (Knoche and Winkler, 2017). High firmness helps ensure post-harvest quality and adequate shelf life (Wani et al., 2014). Among the sweet cherry fruit quality traits, firmness has been rated third in importance by consumers above size and color (Zheng et al., 2016). Likely due to this importance of fruit firmness in the eating experience as well as the increased storability of firm fruit, growers surveyed by Yue et al. (2017) were willing to pay an extra $.55/lb ($1.21/kg) for firmer fruits.

Breeding for increased fruit cracking tolerance and firmness provides a sustained solution in the form of superior new cultivars as opposed to repetitive cultural management practices such as calcium and gibberellic acid applications for both cracking and firmness, which have produced mixed results (Choi et al., 2002; Correia et al., 2018; Winkler and Knoche, 2019). Infusing traditional tree fruit breeding operations with DNA information gained through genomics research and practical tool development can make the breeding process more efficient, accurate, and creative (Peace, 2017). Among other operations, the Pacific Northwest Sweet Cherry Breeding Program (PNWSCBP) currently uses DNA information regularly to aid in selection decisions regarding traits including self-fertility (Haldar et al., 2010) and powdery mildew (Peace et al., 2017). Fruit cracking tolerance and high firmness are also important breeding targets of the PNWSCBP, but currently, DNA information is not used routinely in the breeding process to achieve these targets.

Quantitative trait locus (QTL) analysis is an important step in uncovering the genetic factors contributing to a given trait. However, from a breeding perspective, understanding the genetic contribution of a trait is a means to an end rather than an end itself. Recognizing the ultimate goal of crop improvement does not change the QTL analysis *per se*, but it does contextualize the analysis as part of a larger process (Peace, 2017). Ultimately, the information gained through QTL analyses can later be translated into DNA-based diagnostic assays for trait improvement (Vanderzande et al., 2018). Various QTL discovery approaches exist, with an important emphasis on the nature of the study population, guiding which approach is most appropriate (Peace et al., 2014). Highly structured, pedigree-connected populations are common in sweet cherry breeding programs (Quero-Garcia et al., 2019a). A pedigree-based analysis (PBA) approach, as implemented in the FlexQTL™ software (Bink et al., 2014), has been used successfully to discover the firmness QTLs in sweet cherries (Cai et al., 2019; Calle and Wünsch, 2020) in such prepared, pedigree-connected germplasm (Peace et al., 2014), as well as many other related rosaceous crops (Iezzoni et al., 2020). The discovery of the firmness QTLs was also made possible due to the RosBREED cherry 6K single nucleotide polymorphism (SNP) array that was developed for sweet and sour cherries (Peace et al., 2012). Recently, analytical methods for genome-wide association studies (GWASs) such as FarmCPU (Liu et al., 2016) and BLINK (Huang et al., 2018) were developed to increase power in detecting true associations, as well as to limit false-positives that historically have been problematic for GWASs in structured populations (Zhu et al., 2008).

Both fruit cracking and firmness have been shown to be influenced by genetic factors in the breeding germplasm (Campoy et al., 2014; Piaskowski et al., 2018; Cai et al., 2019; Calle et al., 2020; Calle and Wünsch, 2020; Quero-Garcia et al., 2021). Quero-Garcia et al. (2021) recently estimated the broad-sense heritability (*H*^2^) for three types of cracking—pistillar-end, stem-end, and side. For pistillar-end cracking, often the most prevalent form, the *H*^2^ estimates ranged from 0.608 to 0.905, along with estimates from 0.575 to 0.742 and 0.354 to 0.557 for stem-end and side cracking, respectively. Using multiple bi-parental populations, various methodologies for determining cracking propensity, and recording this phenotypic data over an extended period of time (7–8 years), several cracking QTLs have also been reported, thus, establishing the genetic contribution to this phenomenon (Quero-Garcia et al., 2014, 2019b, 2021). Among the most stable of cracking QTLs discovered, Quero-Garcia et al. (2021) reported a QTL on linkage group (LG) 5 for 7 years using a ‘Regina’ × ‘Lapins’ population that was later concluded to be two linked QTLs. The reported *H*^2^ estimates for fruit firmness, derived from various estimation methods, ranged from 0.77 to 0.85 (Campoy et al., 2014; Piaskowski et al., 2018; Calle and Wünsch, 2020). The narrow-sense heritability (h^2^) for fruit firmness was estimated as 0.27 (Piaskowski et al., 2018). Fruit firmness QTL discoveries also support the underlying genetic contribution to the trait. Firmness QTLs have been detected on all eight chromosomes of sweet cherry (Campoy et al., 2014; Cai et al., 2019; Calle et al., 2020; Calle and Wünsch, 2020). Campoy et al. (2014) reported QTLs on LGs 1, 2, and 5 that were stable for three out of the 6 years they studied, with Calle and Wünsch (2020) detecting a QTL in the same region of LG 1 over 2 years. Additionally, Cai et al. (2019) and Calle and Wünsch (2020) both detected a stable, large-effect QTL on LG 4 that was hypothesized to be involved in domestication (Cai et al., 2019).

Many pedigree connections in sweet cherry are known (Peace et al., 2014; Vanderzande et al., 2019), with additional discoveries being made (Demir, 2019; Howard et al., 2021; Peace, pers. comm.). These pedigree connections are foundational for effective QTL analyses using PBA (Peace et al., 2014), as has been demonstrated in practice (Peace et al., 2017; Cai et al., 2019; Calle and Wünsch, 2020). The confirmation of previously discovered QTLs, as well as the detection of any as-yet-undiscovered QTLs, is necessary for each breeding germplasm, as QTLs are frequently population-dependent (Peace et al., 2014).

The objectives of this work were to detect and characterize fruit cracking and firmness QTLs using the breeding germplasm of the PNWSCBP, as well as to estimate the effects and identify the origins of the functional SNP haplotypes at significant QTLs.

## Materials and Methods

### Plant Material

Unselected seedlings (*n* = 306) from the PNWSCBP at the Washington State University were used for this work, all of which were located at the Roza Research Farm in Prosser, Washington, and managed according to commercial cultural practices. The seedlings were from two populations. RosBREED seedlings (*n* = 149) were a subset of the RosBREED Crop Reference set, representing 23 important breeding parents (IBPs) and was developed specifically for use in QTL discovery *via* a PBA approach (Peace et al., 2014). RosBREED seedlings, planted in 2006–2008 and grown on ‘Gisela 6’ rootstocks, were chosen through a stratified random sample, the strata being defined by the previous firmness classifications by Cai et al. (2019), in order to ensure relatively equal firmness-class representation. The program seedlings (*n* = 157) were a subset of the available PNWSCBP seedlings planted from 2012 to 2014 on their own roots, which increased the representation of the 22 IBPs. The program seedlings were chosen through a simple random sample. Seedlings with at least one undomesticated parent were excluded from the analyses to reduce confounding effects due to population structure, as well as focus on QTL discovery in more immediately breeding-relevant germplasm. This reduced the final number of seedlings used in the analyses to a total of 259 (Supplementary Table 1) (*n* = 119 RosBREED, *n* = 140 Program) representing 22 IBPs with an average allelic representation (Peace et al., 2014) of at least 20, aside from the IBP ‘Ambrunes,’ which had an average allelic representation of 6.5.

### Trait Evaluation

Fruits were harvested from each seedling tree at peak maturity, as determined by subjective in-field observations of skin and flesh color, taste, and firmness. Where possible, enough fruit was harvested from each seedling to provide 50 unblemished, uniform fruits for phenotypic evaluations. Extra fruits (beyond 50) were collected as insurance for any necessarily discarded fruits. The fruits were stored on ice in a cooler during transport from the Roza Research Farm to the Washington State University Irrigated Agriculture Research and Extension Center in Prosser, Washington (∼3 miles), where they were refrigerated at 1°C until phenotypic evaluation (<48 h from harvest to evaluation). Upon evaluation, the fruits were removed from the cooler and allowed to warm to room temperature (∼22°C). The fruits were then counted and inspected for defects and blemished fruits were discarded, while retained fruits were photographed. The same fruits were used for both firmness and cracking assays (in years when measured together). Because the method for measuring fruit firmness was non-destructive, it is unlikely that the firmness measurements taken on the fruit influenced cracking assays. Firmness measurements (g/mm) were taken using the FirmTech2 (BioWorks, Wamego, KS, United States), on one cheek (mediolateral axis) of the 50 fruits, with the average firmness used as an estimate for each seedling. Cracking incidence was measured (% cracking incidence) using a modified, high-throughput version of Christensen’s classic method (Christensen, 1972), in which the fruits were fully submerged in deionized water for a period of 4 h, blotted dry, and the proportion of cracked fruits was determined. The cracking incidence determination in this work did not take into account the location of cracking on the fruit as has been done previously (Quero-Garcia et al., 2021), but rather, a binary evaluation of cracked vs. not cracked was performed. Though cracking location observations admittedly would have been ideal, this was logistically not feasible because of the reduced labor resources available and the large number of seedlings used in the study. Cracking and firmness were evaluated in 2019 and 2020 for the Program seedlings, while cracking was evaluated in 2019 and 2020 and firmness in 2012 (Chavoshi et al., 2014) and 2020 for RosBREED seedlings. Untransformed data were used for all analyses after the preliminary analyses indicated highly similar results between untransformed and arc sine transformed data. Multi-year best linear unbiased predictions (BLUPs) were calculated from the 2 years of phenotypic data using the lme4 package (Bates et al., 2015) in R (R Core Team, 2020). Despite firmness only being measured on the Combined (i.e., RosBREED + Program) seedling population together in 2020, each seedling for which a firmness BLUP was calculated had 2 years of phenotypic data, from either 2012 (RosBREED seedlings) or 2019 (Program seedlings), and 2020. For ease of interpretation, BLUPs are presented as the deviations from the intercept determined by the mixed-effect model. Thus, the same units (% for cracking incidence and g/mm for firmness) were used for interpretation. In addition, because the BLUPs were calculated so as to synthesize the 2 years of phenotypic data into one estimate, for clarity, the term “multiyear” is used in place of BLUPs.

### Genotypic Data

RosBREED seedlings were previously genotyped using the RosBREED cherry 6K SNP array (Peace et al., 2012) and curated according to Vanderzande et al. (2019). The genetic map used and subsequently updated by Vanderzande et al. (2019) was initially described in Klagges et al. (2013). Physical positions were based on the Peach [*Prunus persica* L. (Batsch)] genome v2 (Verde et al., 2017; Vanderzande et al., 2019). DNA was extracted from the program seedlings using a modified version of the protocol described by Edge-Garza et al. (2014), using small desiccation chambers with Drierite^®^ (W.A. Hammond Drierite Co., Ltd., Xenia, OH, United States) and metal ball bearings in place of silica. The program seedling DNA samples were diluted to a concentration between 30 and 100 ng/μl and sent to GeneSeek (Lincoln, NE, United States) for genotyping using the 6+9K cherry SNP array (Vanderzande et al., 2020). The raw data received from GeneSeek were curated using a slight truncation of the workflow outlined by Vanderzande et al. (2019). The full curation workflow was not necessary as the RosBREED seedling data had already been curated (Vanderzande et al., 2019) and thus served as a guide for the Program seedlings’ curation. Vanderzande et al. (2019) determined a robust set of 1,617 SNPs that were used for all the analyses here on the Combined seedlings (*n* = 259) and RosBREED seedlings (*n* = 119). For the program seedlings that were scanned with the 6+9K array, a total of 3,302 robust SNPs (including 1,364 of the previous 1,617—253 of the 1,617 SNPs not being robust in the program seedlings alone) were used when analyzing the program seedlings alone (*n* = 140).

### Quantitative Trait Locus Detection and Association Analyses

Quantitative trait locus (QTL) detection *via* PBA was implemented through the FlexQTL™ software (Bink et al., 2002, 2014) that uses a Bayesian approach. Additive-only (A) and additive and dominance (A + D) models were used for the Combined seedlings (Supplementary Table 1) as well as for the RosBREED and Program seedlings separately (Supplementary Table 2). Additional additive-only runs were completed for the Combined seedlings using a new seed for the Markov chain Monte Carlo (MCMC) simulation as well as a new QTL prior (new prior = 5, original prior = 2) to ensure repeatable results. The QTL regions determined by the initial runs were used to create a reduced genetic map for cracking incidence and firmness, respectively (Supplementary Table 3), in order to eliminate background noise, and additive-only runs (two, with different starting seeds for the MCMC) were completed for the Combined seedling population. Only additive runs were performed due to the similar results gained from the A + D and A models using all the markers. The Combined seedling population was of main interest as the increased number of seedlings enabled greater power in QTL detection, and thus the reduced-map FlexQTL™ runs were performed only on the Combined population.

The maximum number of QTLs was set at 10 for all models, and the Markov chain length was set between 200,000 and 500,000 (with a thinning value of 0.1% for computational efficiency) depending on the run to ensure adequate model convergence. Effective chain sample sizes were ensured to be greater than 100 as outlined by Bink et al. (2014). Detected QTLs with a Bayes Factor (BF) of 2 and above were considered significant; a BF of 2–5 indicated positive evidence, 5–10 indicated strong evidence, and 10 and above indicated decisive evidence for the presence of a QTL (Bink et al., 2014). The narrow-sense heritability of each trait was estimated from the FlexQTL™ output by $\frac{\mathrm{vQTL11}}{\left(\mathrm{vQTL11}+\mathrm{vERR11}\right)}$, where vQTL11 was the QTL genetic variance of the additive-only model and (vQTL11 + vERR11) was the total variance (Topuz, 2020). The mean cracking incidence narrow-sense heritability was calculated as the average heritability estimates from the two FlexQTL™ runs for multiyear cracking using the additive-only model and the reduced genetic map. The mean firmness narrow-sense heritability estimates were calculated in the same manner. The proportion of the phenotypic variance explained (PVE) by each QTL was estimated from the FlexQTL™ output of the additive-only model by $\frac{\mathrm{wAVt1}}{\sigma \text{p}2}$, where wAVt1 was the weighted (by QTL probability) variance explained by the QTL and σ_*p*_^2^ was the total phenotypic variance. Estimates of PVE by QTLs discovered using the reduced genetic map were reported as the average estimates from the two additive-only model runs.

A GWAS was performed using the BLINK algorithm (Huang et al., 2018), which is part of the R package GAPIT (Tang et al., 2016; Wang and Zhang, 2021). BLINK uses two fixed-effects models iteratively. One model selects significantly associated SNP(s) using Bayesian information criteria with the exclusion of any SNPs in high linkage disequilibrium (absolute value of Pearson correlation > 0.7) with said SNP(s). The other model tests each remaining SNP using the accumulating significant SNP(s) as cofactors to limit false positives (Huang et al., 2018). If a strong population structure was present, principal components (PCs) included in a traditional association model could help limit false positives (Price et al., 2006). Only SNPs with a minor allele frequency above 0.01 were included in the analyses. SNPs with a family-wise error rate of *p* < 0.05 (Bonferroni adjustment applied for multiple test correction) were considered significantly associated with the traits. Narrow-sense heritability was estimated as the proportion of additive genetic variance to the total phenotypic variance estimated by the GAPIT package, using the multiyear data for each trait. The PVE by each significant SNP was estimated as the adjusted *r*^2^ value when using the associated SNP as an independent variable in a simple linear regression model carried out in the base stats package of R (R Core Team, 2020). PC analysis (PCA) was performed *via* the prcomp() function of the base ‘stats’ package in R (R Core Team, 2020) using the genotypic information (1,617 SNPs) for the Combined population.

### Haplotype Analysis and Single Nucleotide Polymorphism Haplotype Effect Estimates

Guided by the QTL detection results from both FlexQTL™ and BLINK, haploblocks were chosen for haplotype analysis. Haploblocks were chosen based on the location of the “primary SNP” (PS) from the QTL detection. PSs were designated as such by (1) being significantly associated with trait variation as reported by BLINK in either the Combined population or subpopulation (the latter case only occurring for the stable cracking QTL on LG 5), (2) being within a haploblock that was less than 10 cM from the QTL peak as determined by FlexQTL™, and (3) explaining the most phenotypic variance compared to other significant SNPs in the region. The haploblocks were previously established based on the absence of known historic recombination (Vanderzande et al., 2019). Despite the haploblocks still containing some seedling recombination events, the original haploblocks were used so as to capture a more accurate representation of the genotypic variation at the QTL, as opposed to looking at one or a few SNPs. Haploblocks analyzed at the stable QTLs are described in Supplementary Table 4. The allele of the PS was used to classify any recombinant seedling SNP haplotype as the non-recombinant parental haplotype that shared the same PS allele. For example, if a parent had two haplotypes as follows: (H1) AA**B**AAAA and (H2) AB**A**BBAB (“A” and “B” representing SNP alleles at adjacent loci, PS in **bold**), and a seedling inherited the following haplotype from the parent: AA**B**ABAB, this seedling haplotype would be classified as H1 due to the B allele present at the PS. Where the output phasing from FlexQTL™ was uncertain, manual phasing was attempted using parental haplotypes as guides. Uncertain SNP haplotypes after manual phasing were left unassigned. Only SNP haplotypes that were represented at least five times among the seedlings were used for comparative analyses.

Single nucleotide polymorphism haplotype effect estimates were calculated as the average trait value of the seedlings with said haplotype subtracted from the trait yearly/multiyear average. SNP haplotypes and their associated seedling trait estimate were compared pairwise using a Tukey’s Honestly Significant Difference (HSD) test (for firmness; effectively normally distributed) or a Pairwise Wilcoxon Rank Sum test (for cracking; not normally distributed) to determine significantly different pairwise contrasts (family-wise error rate of *p* = 0.05, Bonferroni correction applied). The SNP haplotypes that were significantly different from at least one other SNP haplotype were considered “functional” SNP haplotypes, while those not significantly different from any other haplotype were not assigned an effect. A maximum of three levels of functional SNPs could be assigned: high, moderate, and low (for cracking or firmness). The ranking of functional SNPs was relational, meaning that the assignment of a given SNP haplotype was somewhat dependent on the assignment of other haplotype assignments. High or low assignments were given to haplotypes with the greatest number of significantly different contrasts between all other SNP haplotypes (whether the assignment was high or low depended on whether the effect was high or low). For example, if the average trait level associated with haplotype H1 was significantly higher than H2, H3, and H4 trait levels, H1 would be assigned as a high (cracking/firmness) haplotype. A moderate effect was assigned in two ways (continuing the above example): (a) to H5 if its associated average trait level was significantly higher than H2 but not H3 or H4, or (b) to H3 if its associated average trait level was significantly higher than H6. Boxplots were created using ggplot2 (Wickham, 2016) and ggpubr (Kassambara, 2020) in R (R Core Team, 2020).

### Single Nucleotide Polymorphism Haplotype Tracking in the Pedigree

The SNP haplotypes of QTL haploblocks were manually tracked through the pedigree to the earliest known source using Microsoft Excel. SNP haplotypes (determined by FlexQTL™ using genotypic data and the known pedigree) at QTL haploblocks for all seedlings and their known ancestors were imported into an Excel worksheet along with pedigree information (Peace et al., 2014; Demir, 2019; Vanderzande et al., 2019; Howard et al., 2021; Peace, unpublished). The SNP haplotypes of the seedlings were manually checked to ensure agreement with their parental origins [outcomes being either identical, missing an allele(s) but easily imputed, recombinant, or unassignable, due to uncertain phasing or multiple missing alleles not easily imputed]. SNP haplotypes were then tracked (using the pedigree information and basic Excel sorting) from the immediate seedling parental generation through subsequent generations, ensuring accuracy, until a terminal ancestor was reached, according to the available pedigree.

## Results

### Trait Distributions, Heritability Estimates, and Correlations

**FIGURE 1:**
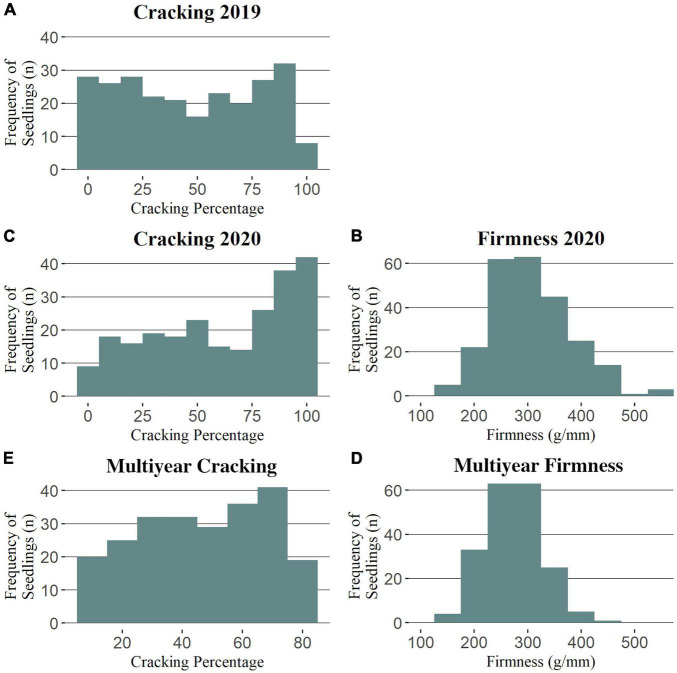
Phenotypic trait distributions for the Combined seedling population. Distributions for cracking incidence in 2019 **(A)**, 2020 **(C)**, and multiyear **(E)** and for firmness in 2020 **(B)** and multiyear **(D)**.

Cracking incidence phenotypic data was successfully collected from a total of 251 and 241 seedlings in 2019 and 2020, respectively (Supplementary Table 5). For fruit firmness, a total of 110 (RosBREED), 140 (Program), and 241 seedlings were successfully evaluated in 2012, 2019, and 2020, respectively (Supplementary Table 6). Seedling cracking incidence was not normally distributed in 2019 nor 2020 (Figure 1), with respective averages of 47 and 61% (Supplementary Table 6). The cracking incidence between subpopulations was not significantly different in either 2019 or 2020 (Supplementary Table 6). Firmness measurements were generally normally distributed (Figure 1 and Supplementary Figure 1), with an average firmness of 309 g/mm in 2020 (Supplementary Table 6), the only year in which all seedlings’ firmness measurements were taken together. Program seedlings were significantly firmer than RosBREED seedlings in 2020, with average firmness values of 330 and 281 g/mm, respectively. The cracking incidence narrow-sense heritability was estimated as 0.34 by FlexQTL™ and 0.54 by GAPIT. The mean firmness narrow-sense heritability was estimated as 0.40 by FlexQTL™ and 0.70 by GAPIT. The seedling firmness levels were significantly correlated between years (2012 vs. 2020 *r* = 0.53; 2019 vs. 2020 *r* = 0.69), as was cracking incidence (2019 vs. 2020 *r* = 0.58) (Supplementary Figure 2). Multiyear firmness and cracking levels were slightly negatively correlated (*r* = −0.1) (Supplementary Figure 2); however, this correlation was not statistically significant.

### Quantitative Trait Loci Detection

The total PVE for all multiyear cracking QTLs detected *via* FlexQTL™ was 24.4% (0.244) and 36.7% (0.367) for all QTLs detected *via* BLINK (Table 1). With the cracking *h*^2^ estimated at 0.34–0.54, the combined multiyear cracking QTLs accounted for 68–72% of the additive genetic variance. The total PVE for all multiyear firmness QTLs detected *via* FlexQTL™ was 30.9% (0.309), and 34.1% (0.341) for all QTLs detected *via* BLINK (Table 1). With the firmness *h*^2^ estimated at 0.4–0.7, the combined multiyear firmness QTLs accounted for 49–77% of the additive genetic variance. The difference between the total PVE by QTLs detected *via* FlexQTL™ and BLINK varied some for both cracking incidence and firmness; the largest variation was in 2019 cracking incidence results. The total PVE for all 2019 cracking QTLs detected *via* FlexQTL™ was 19.7% (0.197), while BLINK did not detect any QTLs (Table 1).

**TABLE 1:** All quantitative trait loci (QTLs) detected from the Combined seedling population using the final reduced map FlexQTL™ analysis or BLINK.

Trait	Year	LG	QTL interval (cM)^a^	Peak position (cM)^b^	Peak position (Mbp)^c^	Peak SNP^d^	Peak SNP position (cM)^e^	Peak SNP position (Mbp)^f^	PVE^g,h^	Name^i^	Cited^j^
Cracking	2019	**1**	**[41, 47]**	**44**	**∼13.5**	–	–	–	**2^e^**	**qCrack-LG1.1m**	1
		**5**	**[0, 37]**	**34**	**∼12.9**	–	–	–	**7.6^e^**	**qCrack-LG5.1m**	1
		7	[49, 74]	55, 57	∼18.4	–	–	–	8.1^e^	qCrack-LG7.2	1
		8	[38, 59]	39, 52	∼14.2, 16.5	–	–	–	2^e^	qCrack-LG8.1m	–
	2020	**1**	**[44, 72]**	**51**	**∼21**	**ss490546566**	**44.95**	**15.1**	**12.6^e^**; **15.1^f^**	**qCrack-LG1.1m**	1
		3	–	–	–	ss490551560	40.87	15.9	4.8^f^	qCrack-LG3.1	1
		**5**	**[31, 53]**	**47**	**∼15**	**ss490559206**	**48.22**	**15.5**	**11.2^e^**; **11.9^f^**	**qCrack-LG5.1m**	1
		7	–	–	–	ss490550511	16.8	10.7	6.2^f^	qCrack-LG7.1	–
		8	–	–	–	ss490558540	59.96	18.1	4.2^f^	qCrack-LG8.1m	1
Multiyear cracking	2019, 2020	**1**	**[44, 55]**	**45**	**∼15.2**	**ss490546611**	**45.51**	**16**	**10.5^e^**; **12.7^f^**	**qCrack-LG1.1m**	1
		3	–	–	–	ss490551560	40.87	15.9	3.6^f^	qCrack-LG3.1	1
		**5**	[2, 41] **[44, 53]**	22, **48**	∼10.2**, 15.5**	**ss490559206**	**48.22**	**15.5**	**8.1^e^**; **13.8^f^**	**qCrack-LG5.1m**	1
		7	[49, 74]	56, 57	∼18.4	–	–	–	3.5^e^	qCrack-LG7.2	1
		8	[48, 62]	55, 56	∼17.3	ss490558540	59.96	18.1	2.3^e^; 6.6^f^	qCrack-LG8.1m	1
Firmness	2020	**1**	**[45, 70]**	**48**	**∼18**	**ss490558902**	**47.55**	**17.6**	**15.6^e^**; **8.8^f^**	**qFirm-LG1.2m**	2, 3, 4
		1	–	–	–	ss490548183	115.11	38	<0.1^f^	qFirm-LG1.3	2
		2	–	–	–	ss490559045	60.16	25.7	<0.1^f^	qFirm-LG2.1	2
		**3**	**[50, 66]**	**62**	**∼21.2**	**ss490551714**	**49**	**18.6**	**7.9^e^**; **10.1^f^**	**qFirm-LG3.2m**	2
		4	[0, 13]	9	∼3.4	ss490552495	8.88	3.4	6.1^e^; <0.1^f^	qFirm-LG4.1	2
		4	–	–	–	ss490552931	34.17	11.5	9.5^f^	qFirm-LG4.2	2, 3, 5
		6	–	–	–	ss490555068	9.75	3.1	9.7^f^	qFirm-LG6.1	–
		6	–	–	–	ss490555531	43.67	9.9	3.4^f^	qFirm-LG6.4	4
Multiyear firmness	2012/2019, 2020	1	–	–	–	ss490545817	13.27	6	2.9^f^	qFirm-LG1.1	–
		**1**	**[34, 70]**	**48**	**∼18**	**ss490546574**	**45.05**	**15.3**	**21.8^e^**; **19.2^f^**	**qFirm-LG1.2m**	2, 3, 4
		3	–	–	–	ss490551234	21.59	4.5	6.0^f^	qFirm-LG3.1	2
		**3**	**[53, 66]**	**62**	**∼21.2**	**ss490551889**	**60.92**	**21**	**9.1^e^**; **1.7^f^**	**qFirm-LG3.2m**	2
		6	–	–	–	ss490555475	30.69	8.6	4.2^f^	qFirm-LG6.2	4
		6	–	–	–	ss490555481	31.64	8.7	0.1^f^	qFirm-LG6.3	4

*LG, linkage group. QTLs in bold type were detected in all years analyzed by FlexQTL™ and at least twice by BLINK. Underlined single nucleotide polymorphisms (SNPs) were designated as “primary” SNPs because they had a higher phenotypic variance explained (PVE) than other BLINK output SNPs at that given QTL in at least 2 years/overall (only determined for stable QTLs).*

*^a^Combined QTL interval from two FlexQTL™ runs (additive model) – interval boundaries indicated.*

*^b^QTL peak position estimate from FlexQTL™ (genetic map estimate) (estimates from both runs included if not identical).*

*^c^QTL peak position estimate from FlexQTL™ (physical map estimate, based on Peach [P. persica L. (Batsch)] genome v2; Verde et al., 2017) (estimates from both runs included if not identical).*

*^d^Top associated SNP (BLINK).*

*^e^BLINK SNP genetic position.*

*^f^BLINK SNP physical position.*

*^g^Phenotypic variance explained (PVE) (average from two runs) estimated from FlexQTL parameters.*

*^h^PVE estimated by adjusted r^2^ value from linear regression.*

*^i^Naming format = [qTrait-LGX^1^.X^2^] where LG, linkage group; X^1^, linkage group indicator; X^2^, order of QTL on LG; m, found in multiple years.*

*^j^1 = Quero-Garcia et al. (2021), 2 = Campoy et al. (2014), 3 = Cai et al. (2019), 4 = Calle et al. (2020), 5 = Calle and Wünsch (2020).*

**FIGURE 2:**
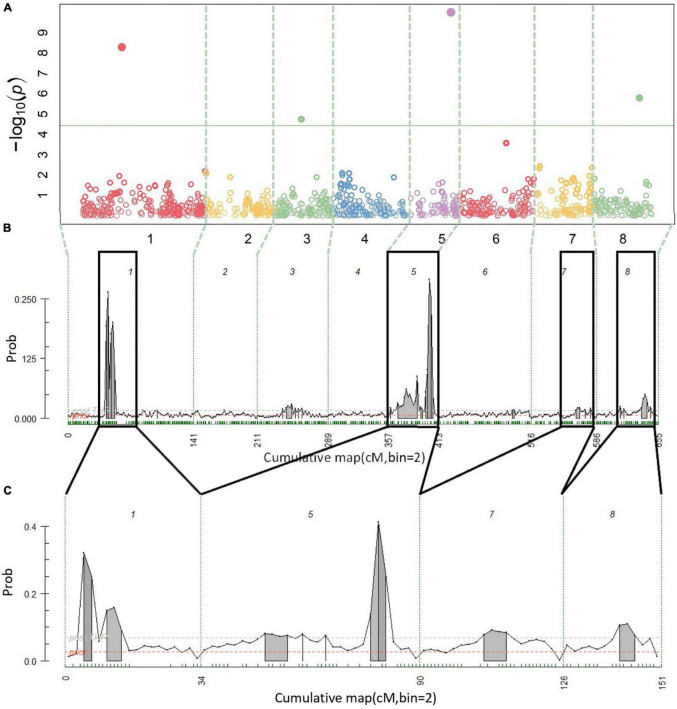
Multiyear cracking incidence quantitative trait locus (QTL) detection results. Manhattan plot from BLINK **(A)**, posterior probabilities of QTL positions from FlexQTL™ full genetic map analysis **(B)**, and FlexQTL™ reduced map analysis **(C)**. Dashed vertical lines **(A–C)** demarcate the chromosomes. Colored circles in **(A)** represent individual single nucleotide polymorphisms (SNPs), their height corresponding to the degree of association between variation in SNP genotype and trait variation. The green horizontal line in **(A)** indicates a Bonferroni adjusted *p*-value of 0.05. Small green dashes along the horizontal axes in **(B)** and **(C)** indicate SNPs grouped in 2 cM bins. Peaks indicate evidence for a QTL at that genomic location, with an area filled in gray indicating positive QTL evidence. The dark black outlines in **(B)** indicate approximate intervals from the full genetic map used to create the reduced map in **(C)**. Results from both FlexQTL™ runs were achieved with an additive-only model.

**FIGURE 3:**
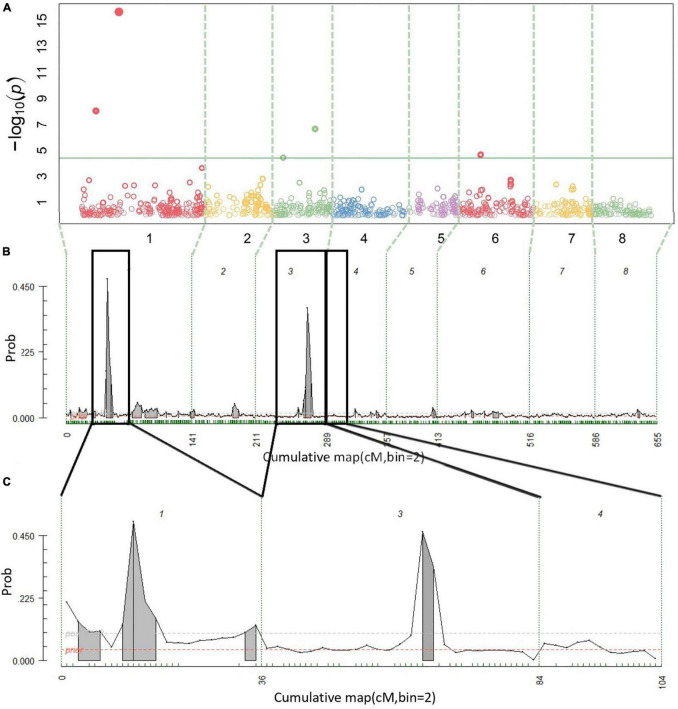
Multiyear firmness QTL detection results. Manhattan plot from BLINK **(A)**, posterior probabilities of QTL positions from FlexQTL™ full genetic map analysis **(B)**, and FlexQTL™ reduced map analysis **(C)**. Dashed vertical lines **(A–C)** demarcate the chromosomes. Colored circles in **(A)** represent individual SNPs, their height corresponding to the degree of association between variation in SNP genotype and trait variation. The green horizontal line in **(A)** indicates a Bonferroni adjusted *p*-value of 0.05. Small green dashes along the horizontal axes in **(B,C)** indicate SNPs grouped in 2 cM bins. Peaks indicate evidence for a QTL at that genomic location, with an area filled in gray indicating positive QTL evidence. Dark black outlines in **(B)** indicate approximate intervals from the full genetic map used to create the reduced map in **(C)**. Results from both FlexQTL™ runs were achieved with an additive-only model.

A total of 14 cracking incidence QTLs were found using FlexQTL™ or BLINK, analyzing the Combined seedling population’s yearly trait data from 2019 and 2020, as well as multiyear cracking incidence (Table 1). Likewise, a total of 14 firmness QTLs were found using the Combined seedling population’s yearly data from 2020 and multiyear firmness (Table 1). The majority of those detected were in regions where QTLs have been previously reported (Table 1). Additional QTLs were detected analyzing the RosBREED and Program subpopulations separately (Supplementary Tables 7–9). For the Combined seedling population, the FlexQTL™ analyses resulted in fewer QTLs detected when compared with BLINK except in the analyses of 2019 and multiyear cracking incidence (Table 1). For multiyear cracking incidence in the Combined seedling population, FlexQTL™ detected QTLs on LGs 1, 5, 7, and 8, and BLINK detected QTLs on LGs 1, 3, 5, and 8 (Figure 2 and Table 1). For multiyear firmness, FlexQTL™ detected QTLs on LGs 1 and 3, whereas BLINK detected QTLs on LGs 1 (two), 3 (two), and 6 (Figure 3 and Table 1). Four QTLs detected by FlexQTL™, two for cracking incidence on LGs 1 (qCrack-LG1.1m) and 5 (qCrackLG5.1m) (Figure 2) and two for firmness on LGs 1 (qFirm-LG1.2m) and 3 (qFirm-LG3.2m) (Figure 3), were stable across years (Supplementary Table 10) and were also found using BLINK (Table 1). These four QTLs were thus the main focus for subsequent analyses. qCrack-LG5.1m and qFirm-LG1.2m were detected in both subpopulations in all years (Supplementary Table 7). qCrack-LG1.1m was detected in the Program seedlings in 2019 and 2020, while it was not detected in either year in the RosBREED seedlings (Supplementary Table 7). Additionally, qFirm-LG3.2m was not detected in the RosBREED subpopulation at all, while it was detected every year in the Program seedling population.

Preliminary results using a compressed mixed-linear model in GAPIT indicated that the use of zero PCs in the model was most effective (highest Bayesian Information Criterion, results not shown); thus, no PCs were used. Exploratory tests incorporating the first two PCs using BLINK still detected the four stable QTLs using multiyear cracking and firmness phenotypic data (results not shown). Although population structure was minimal (the first two PCs explained only ∼18% of variation; Supplementary Figure 3), the possible influence of population structure on BLINK results was explored. Manual incorporation of covariates (two) into BLINK tagged offspring of ‘Ambrunes’ and ‘Sweetheart’ × ‘Sweetheart’ resulted in some QTLs’ associations being diminished, even to the point of non-significance, yet general trends were similar (Supplementary Table 11).

Combining evidence from yearly and multiyear run reports, the cracking incidence QTL on LG 1 (qCrack-LG1.1m) was located at 41–72 cm, peaked at 44–51 cm (∼13.5–21 Mbp), and was estimated to explain just 2% of the 2019 cracking phenotypic variance, 13–15% of the 2020 phenotypic variance, and 11–13% of the multiyear cracking incidence variance (Table 1). The other stable cracking incidence QTL on LG 5 (qCrack-LG5.1m) was located at 0–53 cm, peaked at 22–48 cm (∼10.2–15.5 Mbp), and was estimated to explain 8%, 11–12%, and 8–14% of 2019, 2020, and multiyear cracking phenotypic variance, respectively (Table 1). Ranges in PVE represent estimates by FlexQTL™ and linear regression using significant SNPs, in ascending order.

The firmness QTL on LG 1 (qFirm-LG1.2m) coincided with qCrack-LG1.1m (45–70 cM), peaked at 45–48 cM (∼15.3–18 Mbp), and was estimated to explain 9–16 and 19–22% of the 2020 and multiyear firmness phenotypic variance, respectively (Table 1). The firmness QTL on LG 3 (qFirm-LG3.2m) was located at 49–66 cM, peaked at 49–62 cm (∼18.6–21.2 Mbp), and was estimated to explain 8–10% and 2–9% of the 2020 and multiyear firmness phenotypic variance, respectively (Table 1).

BLINK showed some variability when manually incorporating covariates into the model demarcating offspring of ‘Ambrunes’ and ‘Sweetheart’ × ‘Sweetheart’ (Supplementary Table 11). For example, when analyzing multiyear cracking and firmness phenotypic data, both qCrack-LG5.1m and qFirm-LG3.2m were not significant after the Bonferroni correction was applied (Supplementary Table 11). They were both detected in other years’ data analyses (Supplementary Table 11). Preliminary results indicated that the best model included no PCs, and results from FlexQTL™ aligned well with BLINK results when using no covariates or PCs.

### Quantitative Trait Loci Haplotype Effects

**FIGURE 4:**
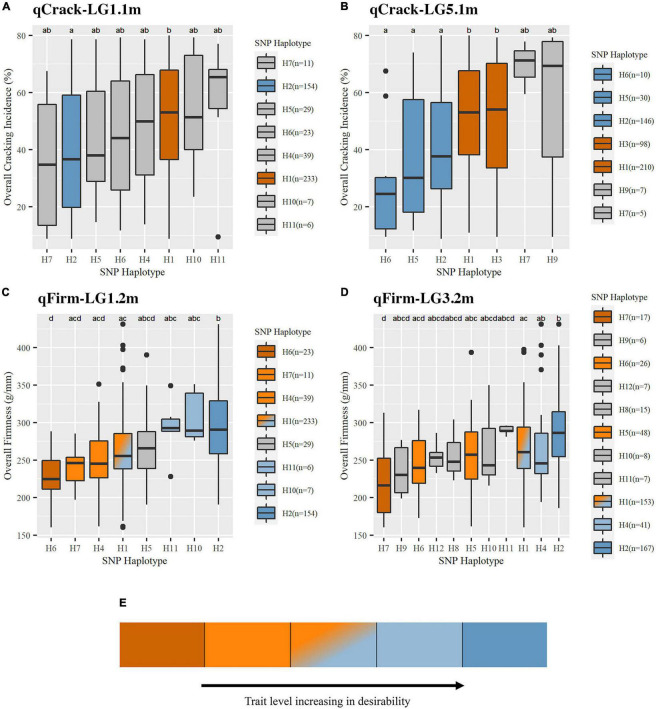
Trait levels associated with SNP haplotypes at the four stable QTLs were detected for cracking incidence **(A,B)** and firmness **(C,D)**. An illustrative description of non-gray coloring used in this figure as well as Table 2 and Supplementary Table 11 **(E)**. Boxes represent the interquartile range (IQR), with the median noted by a horizontal bar in the middle of box. “Whiskers” extend to 1.5 (IQR), with points beyond that noted by individual dots. Non-gray coloring indicated the effect associated with SNP haplotypes. Effect assignments were placed in three categories: high, moderate, and low. Dark orange = high cracking or low firmness (undesirable); light orange, orange-blue fade, light blue = moderate cracking or firmness; dark blue = low cracking or high firmness (desirable). Gray coloring indicates where no putative effect assignment was made. SNP haplotype representation (n) is indicated.

Three segments (haploblocks) within or overlapping the intervals of the four QTLs of interest were used for SNP haplotype analysis (Supplementary Table 4). qCrack-LG1.1m and qFirm-LG1.2m had eight common (represented at least five times) SNP haplotypes, qCrack-LG5.1m had seven, and qFirm-LG3.2m had 11 (Supplementary Tables 12, 13). The distribution of the haplotypes at each QTL was skewed heavily, with the two most represented haplotypes accounting for at least 60% of the total number of haplotypes (Supplementary Table 13) at each QTL. The PSs for qCrack-LG1.1m, qCrack-LG5.1m, qFirm-LG1.2m, and qFirm-LG3.2m were ss490546566, ss490554283, ss490546574, and ss490551714, respectively (Table 1 and Supplementary Table 6). SNP ss490554283 was only reported as significant by BLINK in the RosBREED subpopulation (Supplementary Table 7), yet a simple linear regression yielded a higher PVE by this SNP for the Combined seedling population than by the SNP output by BLINK (ss490559206) in both 2019 and 2020 (not shown). In addition, the haploblock containing ss490559206 condensed some seedlings carrying SNP haplotypes H1, H2, and H6 (of the haploblock containing the PS ss490554283) together, which were significantly different from each other (Figure 4 and Supplementary Table 13). PS ss490551714 was selected instead of ss490551889 for qFirm-LG3.2m because ss490551889 explained less than 2% of the multiyear firmness phenotypic variance (Table 1).

**TABLE 2:** Diplotypes of 22 important breeding parents at each of four stable QTLs.

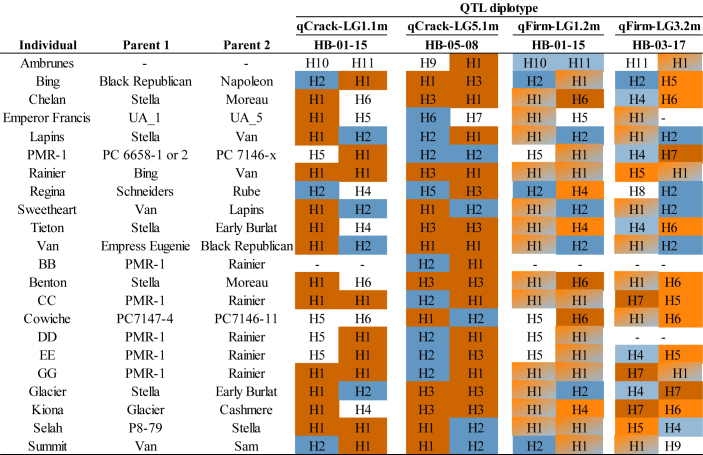

*“HB-XX-XX” indicates the haploblock from which the SNP haplotypes (and diplotypes) were derived. Coloring of haplotypes corresponds to Figure 4E; dark blue indicating either low cracking or high firmness (desirable), light blue, light orange, and orange-blue fade indicating moderate firmness (light blue indicating the SNP haplotype group’s associated phenotype was significantly firmer than dark orange’s, light orange indicating less firm than dark blue, and orange-blue fade indicating both significantly firmer than dark orange but less firm than dark blue), dark orange indicating high cracking or low firmness (undesirable).*

Pairwise comparisons of the effects of common SNP haplotypes using multiyear phenotypic data revealed at least one significant contrast at each of the four QTLs (Figure 4 and Supplementary Table 13). At qCrack-LG1.1m, SNP haplotypes H2 and H1 were associated with significantly different cracking levels and were assigned as putative low-cracking and high-cracking SNP haplotypes, respectively, with H2 associated with a 7% lower multiyear cracking incidence than the population average and H1 associated with a 5% higher multiyear cracking incidence. At qCrack-LG5.1m, H6 (−19%), H5 (−12%), and H2 (−6%) were associated with significantly less cracking than H1 (+4%) and H3 (+5%). Thus, at both multiyear cracking incidence QTLs, two functional SNP haplotype groups were identified—putative low- and high-cracking. At qFirm-LG1.2m, H2 was associated with a significantly firmer fruit than H1, H4, H6, and H7. In addition to being associated with a significantly less firm fruit than H2, H6 was also associated with a less firm fruit than H10 and H11. SNP haplotype H10 for qFirm-LG1.2m was associated with the highest firmness at +37 g/mm, while H6 was associated with the lowest firmness at −40 g/mm (Figure 4 and Supplementary Table 13). At qFirm-LG3.2m, H2 was associated with significantly firmer fruit than H1, H5, H6, and H7, while H1 and H4 were associated with significantly firmer fruit than H7. The most extreme effects were associated with H11 (+20 g/mm) (although not statistically significant, likely due to low representation, *n* = 7) and H7 (−50 g/mm). At both multiyear firmness QTLs, there were three functional SNP haplotype groups; high, moderate, and low firmness (Figure 4 and Supplementary Table 13).

### Single Nucleotide Polymorphism Haplotype Tracking in the Pedigree

All QTL haplotypes for the four QTLs of interest were successfully tracked back to their earliest pedigree source(s) using the known pedigree (Supplementary Tables 13, 14). In addition, QTL diplotypes (combined SNP haplotypes) were successfully compiled for a subset of IBPs (Table 2). ‘Benton,’ ‘Bing,’ ‘Chelan,’ ‘Glacier,’ ‘Kiona,’ ‘Rainier,’ ‘Tieton,’ and ‘Van’ all had two putative high-cracking haplotypes at qCrack-LG5.1m, while ‘Rainier,’ and CC, GG, and ‘Selah’ had two putative high cracking haplotypes at qCrack-LG1.1m. Contrastingly, PMR-1 had two putative low cracking haplotypes at qCrack-LG5.1m (Table 2).

Out of the four possible firmness SNP haplotypes (across the two firmness QTLs), ‘Bing,’ ‘Lapins,’ ‘Regina,’ ‘Sweetheart,’ and ‘Van’ had two putative high-firmness haplotypes, while ‘Benton,’ CC, ‘Chelan,’ ‘Cowiche,’ GG, ‘Glacier,’ ‘Kiona,’ and PMR-1 each had one putative low-firmness haplotype (Table 2). No IBP had both a high- and low-firmness haplotype, while many had either a high- or low-firmness haplotype and a moderate-firmness haplotype (Table 2).

## Discussion

Four stable QTLs, two for fruit cracking and two for fruit firmness, were successfully detected and characterized using a PBA (via FlexQTL™) and a GWAS (via BLINK) in the PNWSCBP germplasm representing 22 IBPs. Putative effect estimates were successfully assigned to SNP haplotypes at each QTL, and these haplotypes were able to be tracked through known pedigree records.

### Trait Distributions, Heritability Estimates, and Correlations

This work attempted to increase the number of individuals studied compared with previous reports, but this strategy came with its own drawbacks, especially in terms of cracking incidence estimates (Quero-Garcia et al., 2021). Quero-Garcia et al. (2021) noted that, when attempting to quantify cracking incidence, a trade-off is inherent in the two, broad phenotyping strategies: (1) improved or refined trait measurement and (2) increased number of individuals. Approximate cracking estimates were achieved here through a modified version of Christensen’s benchtop soaking method (Christensen, 1972); however, this could limit the estimate’s representativeness of a seedling’s cracking propensity in the field. The general agreement in the findings from this work and previous reports gave confidence in the accuracy of the cracking approximations presented here. The non-normal distributions for cracking incidence in both 2019 and 2020 (Figure 1) were consistent with the previous reports (Quero-Garcia et al., 2014, 2021). While such previous studies used arcsine-transformed phenotypic data, preliminary QTL analyses using our untransformed and transformed data had similar outcomes, and the results of the untransformed data were easier to interpret. The greater level of cracking observed in 2020 could have been due to the increased total rainfall in an important fruit growth stage during May 2020 compared to May 2019 (1.78 and 1.32 cm, respectively; AgWeatherNet, 2020), which could have exacerbated fruit microcracking (Knoche and Peschel, 2006) that has been observed to precede macrocracks (Schumann et al., 2019). The effectively normal firmness distributions in all years studied (Figure 1 and Supplementary Figure 1) were also consistent with the previous reports (Quero-Garcia et al., 2014).

The higher narrow-sense heritability estimates (*h*^2^) for both cracking incidence and firmness by GAPIT compared to FlexQTL™ were possibly because GAPIT incorporated all markers to derive its estimate, while FlexQTL™ used only the QTL variance to derive its estimate. A recently reported long-term study estimated the pistillar-end, stem-end, and side cracking *H*^2^ at 0.608–0.905, 0.575–0.742, and 0.354–0.557, respectively (Quero-Garcia et al., 2021). Most of these estimates are higher than the cracking incidence *h*^2^ estimates presented here (0.34–0.54). This discrepancy is to be expected, as broad-sense heritability includes all components of genetic variance. In addition, differences in phenotyping could have influenced differences reported in heritability estimates. The firmness *h*^2^ GAPIT estimate presented here was 0.70, which is similar to previous estimates of broad-sense heritability (*H*^2^) for firmness that has ranged from 0.77 to 0.85 (Campoy et al., 2014; Piaskowski et al., 2018; Calle and Wünsch, 2020). Piaskowski et al. (2018) found that *h*^2^ for firmness was inflated when non-additive variance sources were not included in the model (*h*^2^ jumping from 0.27 in the full model to ∼0.75 in the additive-only model), and this could explain the high *h*^2^ presented here for firmness by GAPIT (a purely additive model). Assuming the genetic model (additive only) is accurate, any missing heritability could be due to many minor-effect QTLs and/or rare alleles that were not detected (Manolio et al., 2009).

Significantly, moderately high between-year correlations for cracking incidence (2019 vs. 2020, *r* = 0.58) and firmness (2012 vs. 2020, *r* = 0.53; 2019 vs. 2020, *r* = 0.69) were comparable to those reported by Quero-Garcia et al. (2014), *r* = 0.49 for stylar cracking from tunnel data and *r* = 0.58 for firmness. Quero-Garcia et al. (2021) recently reported the correlation between fruit firmness and cracking incidence to be quite variable, depending on factors such as germplasm composition, phenotyping methodology, and genotype × environment interactions. It is possible that environmental effects and/or the imperfect phenotyping method for cracking incidence introduced variability in the cracking quantification, thus, obscuring any potential significant correlation. Additional seasons of phenotyping and increasingly expansive methodologies (e.g., in-field quantitative measurements, simulated, and controlled rainfall) in the germplasm presented here could help clarify any putative correlation between firmness and cracking. The germplasm used here is in a particularly advantageous position for this additional phenotyping, as it is part of an active breeding program. The slight negative correlation observed here, while not significant, could be associated with SNP haplotype H2 of qCrack-LG1.1m and qFirm-LG1.2m that was associated with both low cracking and high firmness (Figure 4 and Supplementary Table 13). This location (LG 1, 14.9–17 Mbp, Supplementary Table 4) could be of particular interest to breeders looking to increase cracking tolerance and fruit firmness, as the selection of seedlings (marker-assisted seedling selection), parents (marker-assisted parental selection), or other breeding material (marker-assisted introgression) with putative beneficial alleles for both cracking tolerance and firmness is possible.

### Quantitative Trait Loci Detection

Analyses using the Combined seedling population were more powerful in detecting QTLs than subpopulations by themselves. This increase in power was manifested in that more QTLs were detected using the Combined population for cracking incidence in 2019, 2020, and multiyear as well as for firmness in 2020 and multiyear (Table 1). Intuitively, the power increase makes sense, because as the sample size grows, the power to detect QTLs increases due to increased representation of the various contrasting-effect alleles present in the germplasm (Peace et al., 2014). However, some QTLs were more readily detected in subpopulations. For example, a firmness QTL on LG 4 that was previously reported by Cai et al. (2019) and Calle and Wünsch (2020) was detected in the analyses presented here using the RosBREED population [a subset of that which was used by Cai et al. (2019)] with 2012, 2020, and multiyear firmness data; however, it was detected only in 2020 when using the Combined seedling population (Table 1). This particular example could be due to the putative role this QTL played in domestication (Cai et al., 2019), with a relatively low allele frequency of the unimproved “soft” allele in the Combined population.

While the variability in the BLINK results could indicate some spurious associations if it were the only analysis method used here, the FlexQTL™ results supported results from BLINK obtained when using no covariates or PCs. Additionally, the small proportion of variation accounted for by the first two PCs (∼18%; Supplementary Figure 3) indicates that population structure was not the major driver of phenotypic variation.

The qCrack-LG5.1m detected here by FlexQTL™ agreed with a QTL found by Quero-Garcia et al. (2021) using a single-QTL analysis approach. The QTL reported there explained 14.5–25.8% of the phenotypic variance. The maximum PVE reported here was 13.8% for qCrack-LG5.1m as determined by linear regression using multiyear cracking incidence (Table 1). Differences in these estimates could be due to different populations, as Quero-Garcia et al. (2021) used three biparental populations. Biparental populations could mean fewer segregating alleles. Using a two-linked QTL analysis method, Quero-Garcia et al. (2021) concluded that there were likely two QTLs on LG 5. The multiyear cracking incidence QTL analysis here *via* FlexQTL™ indeed detected evidence for two QTLs in the qCrack-LG5.1m region, as indicated by two intervals having a comparably high probability of a QTL (Table 1 and Supplementary Table 15). However, this could be artefactual, such that, truly, only one QTL was detected here, as there was only one major posterior probability QTL position peak in the FlexQTL™ multiyear cracking incidence QTL analysis output (Figure 2), and the BLINK results indicated just one QTL (Table 1). In addition, yearly QTL intervals overlapped for qCrack-LG5.1m (Table 1), indicating just one QTL. The first QTL peak reported by Quero-Garcia et al. (2021) from the multi-year analysis of two biparental populations was at 4–14.8 cm, near the top of LG 5, which was not detected by the QTL analyses presented here. However, the majority of evidence presented here (22–48 cM; ∼10.2–15.5 Mbp) was near the second QTL peak position described by Quero-Garcia et al. (2021), at 37–48.8 cm (Table 1; Quero-Garcia et al., 2021). Differences in germplasm used could have contributed to the differences between the studies for LG 5. Both QTLs on LG 5 reported by Quero-Garcia et al. (2021) were detected because of heterozygosity only in ‘Regina.’ While ‘Regina’ was well represented in this study, it is possible the QTL allelic contrast at the top of LG 5 was not as stark in this study due to greater allelic variation across the genome compared to the biparental populations used by Quero-Garcia et al. (2021). In addition, phenotyping methodologies could have also contributed to differences in results presented here and those in Quero-Garcia et al. (2021). Extended years of phenotyping as well as multiple methods and on-fruit locational cracking data recorded by Quero-Garcia et al. (2021) could have resulted in the detection of QTLs not discerned by 2 years of cracking incidence approximation *via* Christensen’s method (Christensen, 1972).

Quantitative trait loci (QTLs) close to qFirm-LG1.2m, ranging from 0 to 7 cm (1–4 Mbp) apart, were reported over 2 years by Calle et al. (2020) using an ‘Ambrunes’ × ‘Sweetheart’ seedling population, with the PVE ranging from 12.7 to 22.5%. This PVE is similar to the PVE estimated here for qFirm-LG1.2m of 8.8–21.8% (Table 1). Campoy et al. (2014) and Cai et al. (2019) also reported a QTL on LG 1 near the interval identified in this study, ranging from 0 to 4 cm (1–2 Mbp) away. Campoy et al. (2014) also reported a QTL overlapping with qFirm-LG3.2m; however, it was not stable across multiple years. In addition, Campoy et al. (2014) reported stable QTLs on LGs 2 and 5, which were only detected transiently or not at all, respectively, in this study. It is possible that this QTL on LG 2 and the stable QTL reported by Campoy et al. (2014) on LG 5 were not detected in this germplasm due to differences in allelic composition and/or frequencies. Calle and Wünsch (2020) confirmed the presence of a stable fruit firmness QTL on LG 4, earlier reported by Cai et al. (2019), which was detected here (34 cm, 11.5 Mbp), albeit only inconsistently likely due to differing allelic representation in the germplasm tested.

The between-year variation in PVE by each stable QTL (Table 1) could have been contributed to by environmental variation. Indeed, Quero-Garcia et al. (2021) reported that rainfall amounts were more influential in their model in explaining genotypic differences than were other covariates such as other fruit quality traits. In addition, the greatest disparity in between-year PVE estimates came at qCrack-LG1.1m (PVE = 2% in 2019, 12.6–15.1% in 2020), and this trait has been notoriously difficult to phenotype accurately (Quero-Garcia et al., 2014, 2021). Thus, it is possible that imperfections in the phenotyping methodology used here contributed to differences in PVE between years.

The stable BF value of “NA” for qFirm-LG1.2m derived from the 2020 firmness data using the reduced genetic map (Supplementary Table 10) was unexpected, as the BF using the full genetic map was decisive (BF > 25) (Supplementary Table 8). The values of “NA” were frequent in results from model comparisons in which much higher QTL numbers were compared, indicating those model comparisons were “not applicable.” Thus, the BF value of NA reported here for the model comparison of 1 QTL vs. 0 QTLs at qFirm-LG1.2m could be because there was evidence for two QTLs in this region (Supplementary Table 15), rendering the model comparison of 1 QTL vs. 0 QTLs not applicable. However, as with qCrack-LG5.1m, there is likely only one QTL at qFirm-LG1.2m, because in the two reduced map FlexQTL™ runs, one interval (with a peak at 48 cm; ∼18 Mbp) had the highest posterior probability in both runs, whereas in the first run, an interval downstream had a comparably high (although still lower) probability, and in the second run, an interval upstream had a comparably high (although still lower) probability. Thus, the one interval being stable while others were transient indicates one true QTL in the region. In addition, the BLINK results for this region of LG 1 support the conclusion of one QTL being present in this region in the germplasm studied (Table 1). Calle et al. (2020) concluded that the two QTLs discovered in very close proximity to each other [4 and 7 cm (4 and 7 Mbp) apart in years one and two, respectively] on LG 1 were two different QTLs due to their ostensibly opposite effects, but the authors conceded that the two QTLs could indeed be the same. Evidence presented in this study most strongly supports one firmness QTL on LG 1, yet cannot rule out that there indeed may be others.

Results from the QTL detection in the RosBREED and Program seedling populations separately indicated that some of the QTLs detected in the Combined seedling population were driven disproportionately by one subpopulation. This unequal contribution of subpopulations to the results in the Combined seedling population could be due to the large number of ‘Sweetheart’ × ‘Sweetheart’ seedlings in the Program seedling population. This hypothesis that ‘Sweetheart’ × ‘Sweetheart’ seedlings heavily influence the detection of QTLs was supported by the result that ‘Sweetheart’ was heterozygous at qCrack-LG1.1m, with one putative low-cracking haplotype and one putative high-cracking haplotype (Table 2). The large representation of ‘Sweetheart’ could also explain why qFirm-LG3.2m was not detected in the RosBREED seedling population but was detected in the Program seedling population using 2019, 2020, and multiyear firmness data (Supplementary Table 7). Following the same logic outlined for qCrack-LG1.1m, because ‘Sweetheart’ was also heterozygous at qFirm-LG3.2m with one high-firmness haplotype and one moderate-firmness haplotype (Table 2), the abundance of its offspring could have sharpened the contrast.

### Quantitative Trait Loci Haplotype Effects

Genotypic variation present at the four stable QTLs was successfully characterized *via* SNP haplotype analysis (Supplementary Table 13). Haplotypes, i.e., strings of alleles inherited together, increased the available variation that could be captured as compared with any single SNP. In this sense, haplotypes can be more informative than a single SNP (Flanagan and Jones, 2019).

Kostick et al. (2021) determined the effect of SNP haplotypes by comparing the average trait levels of individuals with a given haplotype against all individuals without the given haplotype. The approach employed in this study was more conservative, as comparisons were performed between a given haplotype and all other haplotypes, which constituted more hypothesis-testing and thus, a more severe (smaller) group-wise significance threshold. By not including haplotype groups with less representation than five, this overly strict significance threshold was remedied to an extent. Comparing each haplotype’s associated trait level against every other haplotype provided a level of granularity in which a “moderate” functional effect could be assigned. Several haplotypes were not considered “functional” (Figure 4). In conjunction with the strict group-wise significance threshold, this lack of functional assignment could be because haplotypes at a given QTL haploblock varied in representation—usually, two haplotypes were highly represented (Supplementary Table 13). The lack of a functional haplotype assignment given here does not preclude that these haplotypes would have a significant effect in other germplasm with a greater representation of the contrasting QTL alleles. Rather, with the current population, no putative effect assignment could be made because no statistical difference was detected between the contrasting haplotypes’ associated trait levels.

### Single Nucleotide Polymorphism Haplotype Tracking in the Pedigree

Similar to how the use of phased data from a fingerprinting panel of neutral linked SNPs can elucidate identity-by-descent and thus confirm pedigree connections (Peace et al., 2017), the ability to track a QTL haplotype from offspring back to the earliest known generation confirms the identity of that haplotype among relatives. Tracking SNP haplotype H2 at qCrack-LG1.1m back from the parent ‘Sweetheart’ to ‘Lapins’ to ‘Van’ to ‘Black Republican’ to an unknown parent (“Unknown Parent of Black Republican”) and finally, an unnamed grandparent of ‘Black Republican’ (“Unnamed Ancestor_1”) that was an ancestor of other germplasm through other lineages (Supplementary Table 1) gave confidence that this haplotype represented the same co-inherited block with its embedded QTL allele.

Considering all four stable QTLs identified in this study, ‘Regina’ or PMR-1 could be used as parents if trying to generate a high proportion of seedlings with a low cracking incidence as they both had two putative low-cracking haplotypes across the two cracking incidence QTLs and only one putative high-cracking haplotype (Table 2). In addition, ‘Emperor Francis’ could be beneficial to increase low-cracking allelic diversity, as it was detected to have a less-frequent putative low-cracking SNP haplotype H6 at qCrack-LG5.1m (Table 2). Conversely, ‘Chelan’ carried three putative high-cracking haplotypes as well as the one low-firmness haplotype at qFirm-LG1.2m, and thus, it may not be an ideal parent if either low cracking incidence or high firmness is of importance in the offspring. Individuals with two putative high-firmness haplotypes, such as ‘Bing,’ ‘Lapins,’ ‘Regina,’ ‘Sweetheart,’ and ‘Van,’ could serve as promising parents for families focused on high firmness, although if intercrossed there would be no allelic diversity contributing to high firmness at these two QTLs (Table 2). The opportunity exists in both cracking and firmness to increase the number of desirable alleles in the breeding material. With two stable, bi-allelic QTLs identified here for each trait, there is a maximum of four desirable alleles for each trait, but among the IBPs studied here, the maximum number of desirable alleles observed was only two (Table 2).

Some seedlings examined had accumulated several putative beneficial SNP haplotypes that could render them useful as parents. Five seedlings had the maximum four putative low-cracking haplotypes (Supplementary Table 12) (their average cracking incidence was 15%, much lower than the population average of 47%; Supplementary Table 6), while seven seedlings had the maximum four putative high-firmness haplotypes (Supplementary Table 12) (with an average firmness of 341 g/mm, 71 g/mm higher than the population average of 270 g/mm; Supplementary Table 6). Based on these results as well as phenotypic data, one seedling with three putative low-cracking and three putative high-firmness haplotypes was used as a female parent in the PNWSCBP in spring 2021 crossing, with nine seeds obtained (McCord, pers. comm.).

## Conclusion

The Combined population of unselected seedlings was the most effective for detecting significant QTLs, yielding more QTLs than either subpopulation alone. Results from FlexQTL™ and BLINK were largely in agreement for stable QTLs and using both analyses gave confidence in results. Considering the results of the QTL analyses of the Combined population in the context of subpopulation QTL analyses was helpful to hypothesize which individuals are driving the phenotypic contrasts associated with each QTL, such as seedlings of ‘Sweetheart’ × ‘Sweetheart’ contributing largely to qCrack-LG1.1m and qFirm-LG3.2m. The multiyear PVE by the combined QTLs detected for fruit cracking incidence and firmness was approximately 24–37 and 31–34%, respectively. Four QTLs, two per trait, were the main genetic factors explaining additive genetic components of that variation and are therefore of breeding relevance. Future research is warranted to translate these findings into practical breeding tools. The results from this study can be and have already been applied practically in a breeding program through parental selection informed by the detected QTLs and their characterized haplotypes.

## Data Availability Statement

The original contributions presented in the study are included in the article/Supplementary Material, further inquiries can be directed to the corresponding author. The datasets (phenotypic and genotypic) and QTL information generated for and by this study can be found in the Genome Database for Rosaceae (rosaceae.org) with accession number tfGDR1053.

## Author Contributions

WC designed and carried out the experiments and analyses, as well as wrote the manuscript. PM designed and oversaw the experiments and analyses and revised the manuscript. CP and ZZ guided the analysis methods and revised the manuscript. All authors contributed to the article and approved the submitted version.

## Conflict of Interest

The authors declare that the research was conducted in the absence of any commercial or financial relationships that could be construed as a potential conflict of interest.

## Publisher’s Note

All claims expressed in this article are solely those of the authors and do not necessarily represent those of their affiliated organizations, or those of the publisher, the editors and the reviewers. Any product that may be evaluated in this article, or claim that may be made by its manufacturer, is not guaranteed or endorsed by the publisher.
