# Supplementary figures and images for: Detection of Breeding-Relevant Fruit Cracking and Fruit Firmness Quantitative Trait Loci in Sweet Cherry via Pedigree-Based and Genome-Wide Association Approaches
Source: Front Plant Sci. 2022 Mar 2;13:823250. doi: 10.3389/fpls.2022.823250 (PMC8924583; doi:10.3389/fpls.2022.823250)

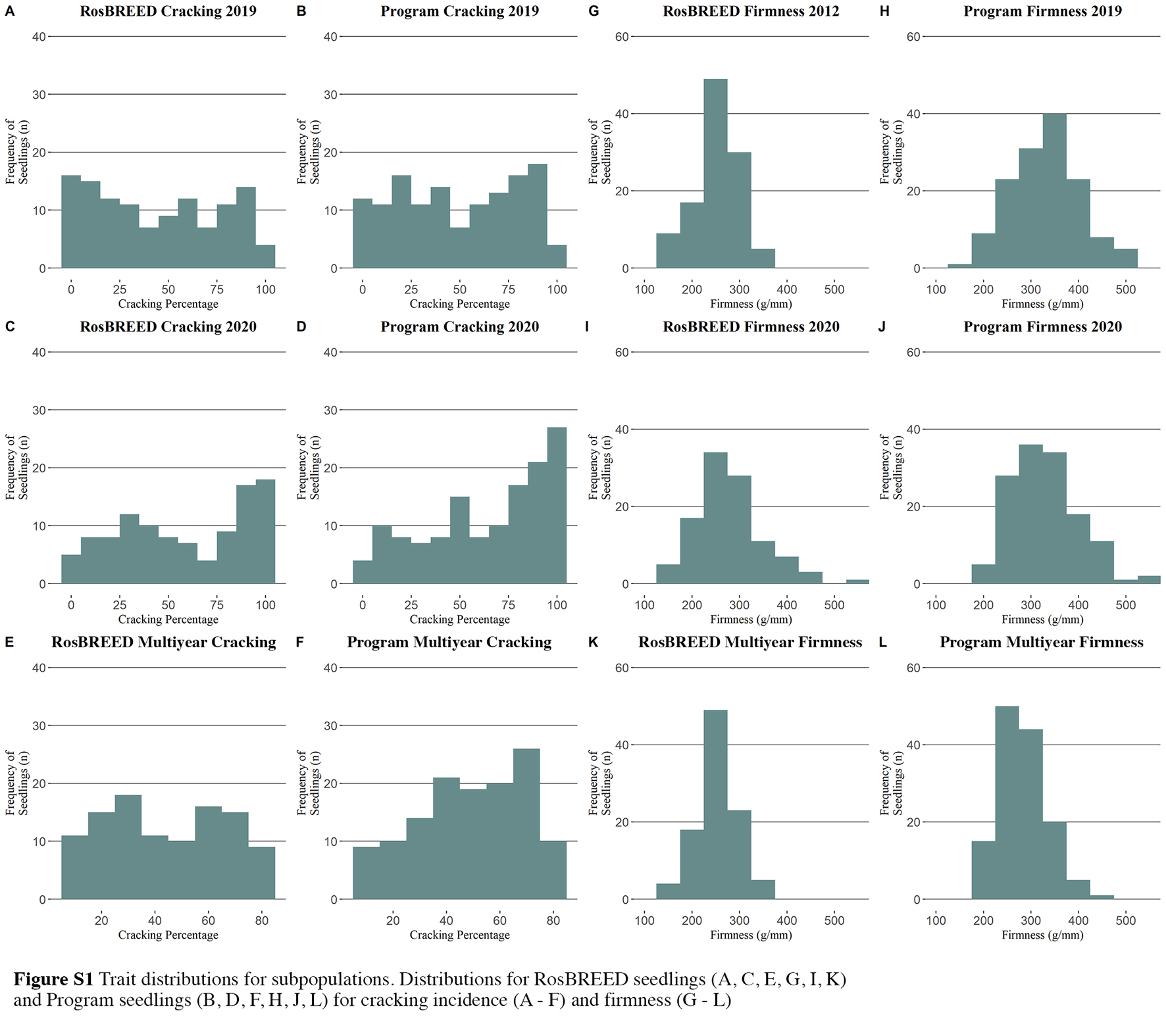

Supplement: Supplementary file 1 [file Image_1.JPEG]

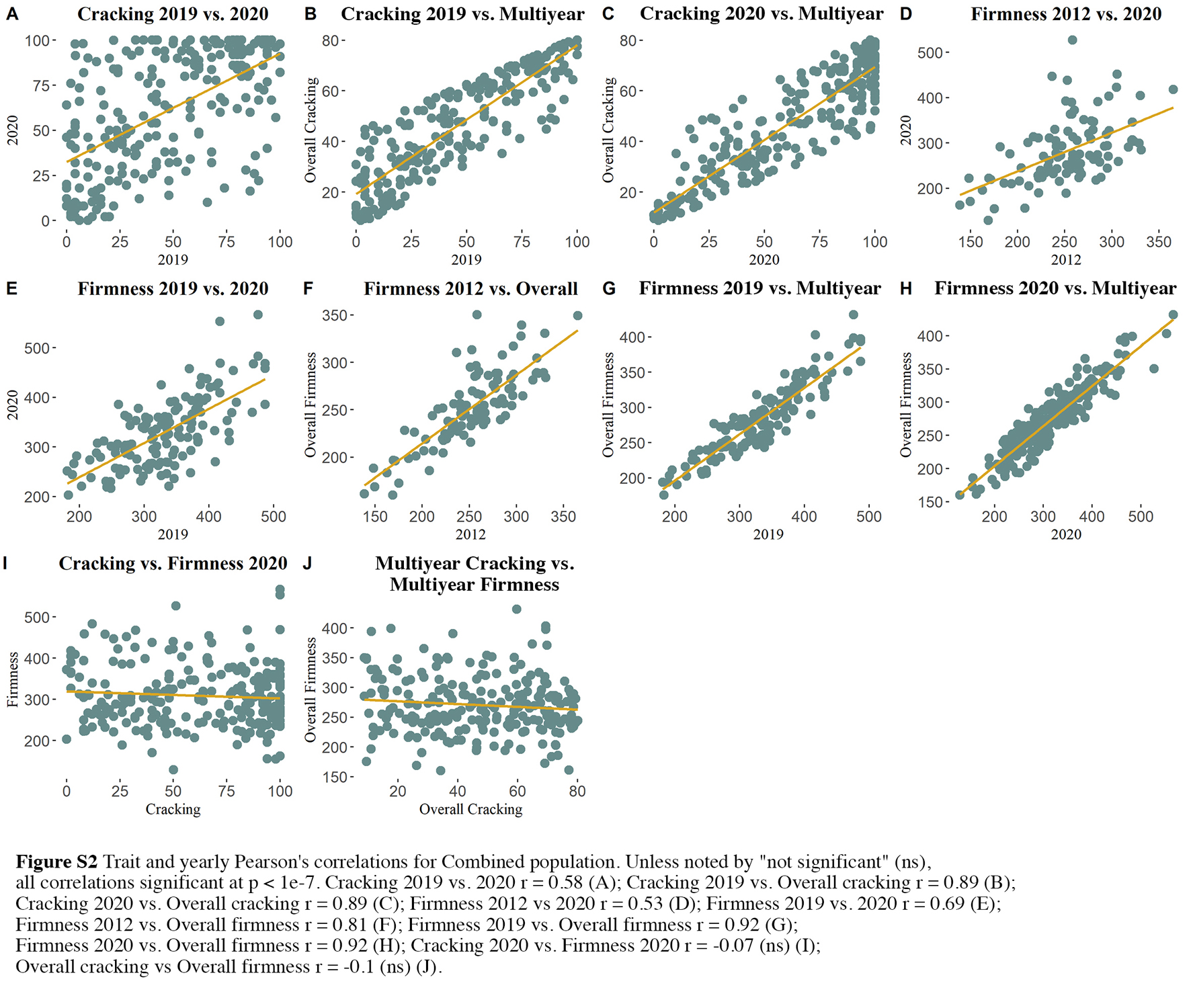

Supplement: Supplementary file 2 [file Image_2.JPEG]

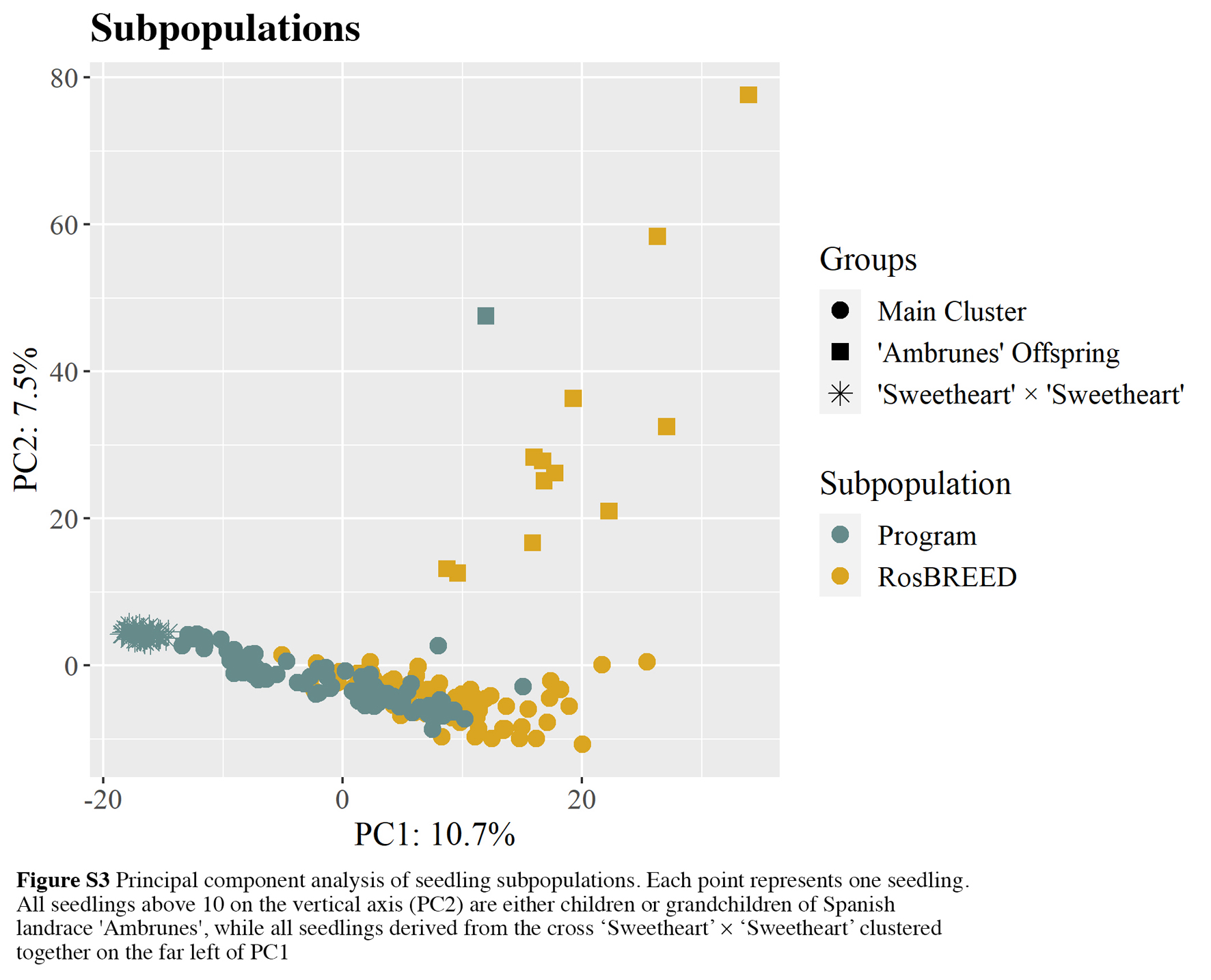

Supplement: Supplementary file 3 [file Image_3.JPEG]
